# Association Between Alcohol Consumption, Other Healthy Habits and Sociodemographic Variables and the Values of Different Insulin Resistance Risk Scales in 139,634 Spanish Workers

**DOI:** 10.3390/healthcare13080921

**Published:** 2025-04-17

**Authors:** Joan Obrador de Hevia, Ángel Arturo López-González, José Ignacio Ramírez-Manent, Hernán Paublini, Pedro Juan Tárraga López, Cristina Martorell Sánchez, Pere Riutord-Sbert

**Affiliations:** 1 ADEMA-Health Group, University Institute of Health Sciences (IUNICS), 07120 Palma, Spain; j.obrador@eua.edu.es (J.O.d.H.); joseignacio.ramirez@ibsalut.es (J.I.R.-M.); h.paublini@eua.edu.es (H.P.); c.martorell@eua.edu.es (C.M.S.); pereriutord@gmail.com (P.R.-S.); 2 Faculty of Dentistry, ADEMA-UIB University School, 07009 Palma, Spain; 3 Balearic Islands Health Service, 07003 Palma, Spain; 4 Balearic Islands Health Research Institute Foundation (IDISBA), 07004 Palma, Spain; 5 Faculty of Medicine, University of the Balearic Islands, 07122 Palma, Spain; 6 Faculty of Medicine, University of Castilla-La Mancha, 02071 Albacete, Spain; pjtarraga@sescam.jccm.es

**Keywords:** insulin resistance, alcohol consumption, Mediterranean diet, physical activity, tobacco consumption, sociodemographic variables

## Abstract

**Background**: Alcohol consumption is a major public health concern, influencing metabolic health and insulin resistance (IR). While moderate alcohol intake has been associated with potential metabolic benefits, excessive consumption is linked to IR and related disorders. This study examines the association between sociodemographic variables, health habits, and IR risk using validated metabolic indices. **Methods**: A dual-phase study was conducted, including a cross-sectional analysis of 139,634 Spanish workers and a retrospective longitudinal follow-up of 40,431 participants (2009–2019). Data on sociodemographic factors (age, sex and socioeconomic status) and health habits (smoking, alcohol consumption, diet and physical activity) were collected through standardized occupational health assessments. IR risk was assessed using the Triglyceride-Glucose Index (TyG), Metabolic Score for Insulin Resistance (METS-IR), and Single-Point Insulin Sensitivity Estimator (SPISE-IR). Binary logistic regression was used for statistical analysis. **Results**: Age, male sex, lower socioeconomic status, smoking, alcohol consumption, physical inactivity and low adherence to the Mediterranean diet were significantly associated with higher IR risk across all indices (*p* < 0.001). Alcohol consumption exhibited a dose-dependent relationship with IR, with excessive intake significantly increasing the risk of IR. Longitudinal data revealed a worsening IR profile over time, particularly among older, low-income and physically inactive individuals. **Conclusions**: Sociodemographic factors and lifestyle habits strongly influence IR. Preventive strategies focused on reducing alcohol consumption, smoking cessation and promoting physical activity and dietary improvements are essential to mitigate the risk of IR, especially in vulnerable populations. Further longitudinal studies are needed to establish causal relationships and refine intervention strategies.

## 1. Introduction

Alcohol consumption represents a significant public health concern worldwide [1], with patterns of intake varying across different populations and cultural contexts [2]. While some studies suggest potential cardioprotective effects from moderate alcohol consumption, particularly in reducing the risk of coronary artery disease, excessive and prolonged alcohol intake is associated with numerous adverse health outcomes [3]. The burden of alcohol-related morbidity and mortality is substantial [4], affecting not only the cardiovascular system [5] but also metabolic processes [6], liver function [7] and neurological health [8]. The World Health Organization (WHO) estimates that alcohol consumption contributes to approximately 3 million deaths annually [9], accounting for a considerable proportion of global disease burden [10]. The harmful effects of alcohol extend beyond individual health, influencing social, economic and occupational domains.

From an epidemiological standpoint, alcohol consumption varies significantly between populations, with factors such as socioeconomic status [11], genetic predisposition [12] and cultural norms [13] playing crucial roles in determining drinking patterns. While certain populations exhibit lower rates of alcohol consumption due to religious or societal restrictions [14], others demonstrate high levels of consumption, particularly in high-income countries. A growing body of research highlights the strong association between chronic alcohol intake and the development of metabolic disorders, including insulin resistance (IR) [15], type 2 diabetes mellitus (T2DM) [16] and obesity [17]. Despite some paradoxical findings suggesting that moderate alcohol consumption may improve insulin sensitivity in certain cases, excessive intake disrupts glucose homeostasis and exacerbates IR [18].

Concurrently, IR has emerged as a major contributor to the global rise in metabolic syndrome, T2DM and cardiovascular diseases [19]. It is characterized by the diminished ability of peripheral tissues, particularly skeletal muscle, liver and adipose tissue, to respond adequately to insulin signaling. This pathological condition leads to compensatory hyperinsulinemia, increased hepatic glucose production and subsequent hyperglycemia [20]. The interplay between alcohol consumption and IR remains a critical area of investigation, as both conditions share common metabolic pathways and risk factors. Understanding the mechanisms underlying these associations is essential for developing targeted preventive strategies and mitigating the global burden of metabolic diseases.

Alcohol consumption is a prevalent lifestyle factor that significantly influences global health outcomes. The epidemiology of alcohol use demonstrates considerable heterogeneity based on demographic, geographic and socioeconomic factors [21]. Data from the WHO indicate that approximately 25% of adults worldwide consume alcohol, with variations in intake patterns between regions. High-income countries tend to exhibit greater per capita alcohol consumption, whereas lower-income regions often experience alcohol-related harm at disproportionately higher rates due to limited healthcare resources and prevention strategies [10].

Excessive alcohol consumption is implicated in the pathogenesis of numerous chronic diseases. Cardiovascular disorders, including hypertension [22], atrial fibrillation [23] and cardiomyopathy [24], are strongly linked to chronic alcohol intake. Moreover, alcohol exerts deleterious effects on metabolic health by promoting visceral adiposity [25], dyslipidemia [26] and systemic inflammation [27]. The liver, being the primary site of alcohol metabolism, is particularly vulnerable to alcohol-induced injury, with conditions such as alcoholic fatty liver disease (AFLD), alcoholic hepatitis and cirrhosis being direct consequences of prolonged excessive drinking [28].

Beyond its metabolic effects, alcohol consumption is a leading cause of injury-related mortality, including road traffic accidents [29], suicides and interpersonal violence [30]. Neuropsychiatric disorders, such as alcohol dependence and alcohol-induced cognitive impairment, further exacerbate the societal and economic burden of alcohol-related harm [31]. Importantly, alcohol consumption has been identified as a major carcinogenic factor, contributing to malignancies of the liver, esophagus, breast and colorectum [32].

From a dose-response perspective, research indicates that the relationship between alcohol intake and health outcomes follows a J-shaped curve. While moderate alcohol consumption, particularly of polyphenol-rich beverages such as red wine, has been associated with certain cardiometabolic benefits, heavy and binge drinking patterns negate these potential advantages and significantly increase disease risk [33]. The concept of “safe” alcohol consumption remains controversial, as emerging evidence suggests that even low-to-moderate intake may contribute to adverse health outcomes over time.

IR is a pathological state in which target tissues exhibit reduced responsiveness to insulin signaling, leading to metabolic dysregulation. Under normal physiological conditions, insulin facilitates glucose uptake by peripheral tissues, suppresses hepatic glucose production and modulates lipid metabolism. However, in insulin-resistant states, these processes become impaired, resulting in hyperinsulinemia and chronic hyperglycemia [34].

The pathophysiology of IR is multifactorial, involving complex interactions between genetic predisposition, lifestyle factors, and inflammatory pathways [35]. Adipose tissue dysfunction plays a central role, as excess visceral adiposity leads to increased secretion of pro-inflammatory cytokines [36], such as tumor necrosis factor alpha (TNF-α) and interleukin-6 (IL-6) [37], which interfere with insulin receptor signaling. Moreover, elevated levels of free fatty acids (FFAs) contribute to lipotoxicity, mitochondrial dysfunction and endoplasmic reticulum stress, further exacerbating IR [38].

At the molecular level, IR is characterized by defects in the insulin signaling cascade. The insulin receptor substrate (IRS)-phosphatidylinositol 3-kinase (PI3K)-Akt pathway is crucial for mediating insulin’s metabolic effects [39]. Disruptions in this pathway result in decreased glucose transporter type 4 (GLUT4) translocation to the cell membrane, impairing glucose uptake in skeletal muscle [40]. Additionally, IR leads to increased hepatic gluconeogenesis and reduced suppression of lipolysis in adipose tissue, contributing to hyperglycemia and dyslipidemia [41].

Beyond metabolic implications, IR is a key driver of endothelial dysfunction, oxidative stress and chronic low-grade inflammation, all of which are implicated in the pathogenesis of cardiovascular disease [42]. Epidemiological studies have demonstrated a strong association between IR and an increased risk of atherosclerosis, myocardial infarction and stroke. Given its far-reaching impact on health, IR is considered a major therapeutic target for preventing metabolic syndrome and its associated complications [43].

The clinical consequences of IR extend beyond glucose dysregulation and encompass a wide range of systemic effects. The strong link between IR and T2DM underscores its significance in metabolic disease progression. Insulin-resistant individuals often develop compensatory hyperinsulinemia, which, over time, leads to pancreatic β-cell dysfunction and overt diabetes [44]. Additionally, IR is closely associated with non-alcoholic fatty liver disease (NAFLD), a condition that shares common pathological features with AFLD. Both NAFLD and AFLD contribute to hepatic steatosis, inflammation and fibrosis, ultimately increasing the risk of cirrhosis and hepatocellular carcinoma [45].

The interplay between alcohol consumption and IR remains an area of active research. Chronic excessive alcohol intake disrupts glucose metabolism by impairing insulin signaling, promoting hepatic IR and altering lipid homeostasis. Ethanol metabolism generates reactive oxygen species (ROS), contributing to oxidative stress and mitochondrial dysfunction, both of which are implicated in IR [46]. Furthermore, alcohol consumption influences gut microbiota composition, leading to increased intestinal permeability and systemic inflammation, two key factors in metabolic dysregulation [47].

Interestingly, some epidemiological studies suggest a potential protective effect of moderate alcohol consumption for insulin sensitivity, possibly mediated by improvements in adiponectin levels and endothelial function. However, these benefits are largely outweighed by the negative consequences of excessive drinking, which include increased visceral adiposity, dyslipidemia and pancreatic dysfunction [48].

Alcohol consumption and IR are two interrelated metabolic factors that significantly impact global health. While moderate alcohol intake has been linked to certain metabolic benefits, chronic excessive consumption contributes to IR, metabolic syndrome and cardiovascular disease. The complex relationship between these conditions underscores the importance of public health interventions aimed at reducing alcohol-related harm and promoting metabolic health. Future research should focus on elucidating the precise mechanisms linking alcohol consumption to IR and identifying effective strategies for mitigating their adverse effects.

The aim of this study is to determine how different sociodemographic variables such as sex, age and socioeconomic status and health habits such as tobacco and alcohol consumption, physical activity and the Mediterranean diet are associated with the values of IR risk scales.

## 2. Materials and Methods

A dual-phase study was conducted, comprising an initial descriptive cross-sectional analysis of 139,634 workers from various regions of Spain representing nearly all employment sectors (56,352 women and 83,282 men). The participants were selected from individuals undergoing routine occupational health assessments within the participating companies.

Subsequently, a retrospective longitudinal study was performed on a subset of the original cohort consisting of 40,431 workers (24,229 men and 16,202 women), covering the period from 2009 to 2019.

All collected data—including analytical, anthropometric and clinical parameters—were obtained by healthcare professionals affiliated with the participating companies. Prior to data collection, standardized protocols were implemented to minimize inter-observer variability.

The study established the following inclusion criteria:

- Age range between 18 and 69 years (i.e., within the working-age population).
- Active employment in one of the participating companies and absence of temporary incapacity at the time of data collection.
- Availability of all necessary variables to calculate the different cardiovascular risk scores.
- Willingness to participate in the study and provide consent for data usage in epidemiological research.
- For the retrospective longitudinal study, availability of complete data for both 2009 and 2019, with no changes in socio-demographic characteristics or health-related behaviors during this period.

**Figure 1:**
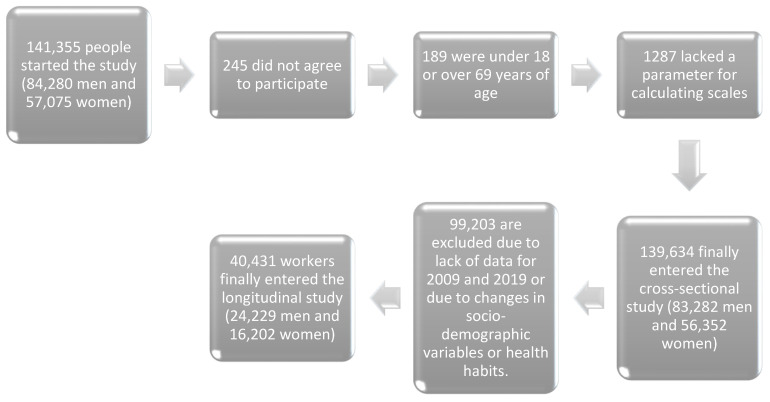
Flow chart of the participants.

The selection process for study participants is illustrated in the corresponding flowchart (Figure 1).

### 2.1. Determination of Variables

The collection of clinical, analytical and anthropometric data necessary for determining an individual’s heart age was conducted by health professionals affiliated with the participating companies using the ISAK criteria [49]. To minimize interobserver variability, all measurements were standardized. Waist circumference was assessed using a tape measure positioned at the level of the last rib, with the participant standing and their abdomen relaxed. Blood pressure was measured using an OMRON M3 sphygmomanometer (OMRON, Osaka, Japan) after the participant remained seated at rest for 10 min. Three consecutive measurements were taken, and the average value was recorded.

Biochemical analyses were performed following a 12 h fasting period. The high-density lipoprotein (HDL) cholesterol level was assessed using precipitation techniques, whereas enzymatic methods were applied for the quantification of blood glucose, triglycerides and total cholesterol levels. Low-density lipoprotein (LDL) cholesterol concentrations were estimated indirectly using the Friedewald Equation [50]. All biochemical parameters were reported in mg/dL.

The evaluation of IR was conducted using the following validated risk indices:

- Triglyceride-Glucose Index (TyG) [51]: Computed using the formula TyG = LN (triglycerides × fasting glucose/2), where values equal to or exceeding 8.5 indicate a high risk of insulin resistance. An extended version, TyG-BMI, incorporates the body mass index (BMI) and is calculated as TyG × BMI.
  The Triglyceride-Glucose Index (TyG) has demonstrated adequate validity as an indirect marker of insulin resistance, showing a strong correlation with the reference method HOMA-IR and other clinical measures. Regarding its reliability, studies assessing its internal consistency using Cronbach’s alpha have reported values above 0.80, indicating good reliability. These findings support its utility as an accessible, practical and reliable tool in both clinical and epidemiological contexts.
- Single-Point Insulin Sensitivity Estimator (SPISE): Derived using the equation SPISE = (600 × HDL^0.185^/(triglycerides^0.2^ × BMI^1.338^) and its inverse, the SPISE-IR, which is calculated as 10/SPISE. An SPISE-IR value of 1.51 or greater is indicative of elevated insulin resistance risk [52].
  The SPISE has shown good validity for estimating insulin sensitivity, particularly in pediatric and adolescent populations, demonstrating adequate correlation with reference methods such as the euglycemic clamp. In terms of reliability, studies evaluating its internal consistency using Cronbach’s alpha reported values close to or above 0.80, supporting its use as a reliable, simple and noninvasive tool for assessing metabolic risk in various clinical and population settings.
- Metabolic Score for Insulin Resistance (METS-IR) [50]: Determined using the formula METS-IR = LN(2 × glucose) + (triglycerides × BMI)/LN(HDL-c). A threshold of 50 or above is considered high risk for insulin resistance [53].
  The METS-IR has demonstrated high validity for estimating insulin resistance, showing a strong correlation with the euglycemic clamp method and HOMA-IR. Furthermore, it exhibits good discriminative capacity for identifying metabolic risk across diverse populations. Regarding reliability, studies assessing its internal consistency using Cronbach’s alpha have reported values above 0.80, indicating adequate stability and reliability for clinical and epidemiological applications.

Participants were classified as smokers if they had consumed at least one cigarette per day (or its equivalent in other forms of tobacco use) within the preceding 30 days or if they had ceased smoking within the last year.

Adherence to a cardioprotective diet was assessed using the “Mediterranean Diet Adherence Questionnaire” from the PREDIMED study. This tool comprises 14 items, each scored on a binary scale (0–1). A score of 9 or above is indicative of strong adherence to the Mediterranean diet, reflecting a heart-healthy dietary pattern [54].

Physical activity levels were evaluated using the International Physical Activity Questionnaire (IPAQ), which assesses activity levels over the previous week. The IPAQ is structured in three main sections: physical activity at work, transportation and recreational activities. It assesses the frequency, duration and intensity of physical activities performed over a week, classifying them as vigorous, moderate or walking. Additionally, it includes a section on sitting time. The results are expressed in MET minutes per week, allowing the estimation of physical activity levels and comparison between individuals and populations. It is widely used in epidemiological and public health studies [55].

Alcohol consumption was quantified in standard alcohol units (SAUs), where one SAU corresponds to 10 g of pure ethanol, as per Spanish guidelines. High alcohol intake was defined as weekly consumption of ≥14 SAUs for women and ≥21 SAUs for men [56].

Occupational social class was determined following the 2011 National Classification of Occupations (CNO-11) and the criteria established by the Spanish Society of Epidemiology [57]. Participants were categorized into three groups:

- Class I: University professionals and senior managers.
- Class II: Skilled self-employed workers and intermediate-level occupations.
- Class III: Unskilled workers.

Educational attainment was classified into three levels: primary education, secondary education, and university education.

### 2.2. Statistical Analysis

For the quantitative variables, mean values and standard deviations were calculated using the Student’s *t*-test. The chi-squared test was applied to determine the prevalence of categorical variables. A multinomial logistic regression model was employed for multivariate analysis. Statistical analyses were conducted using SPSS version 29.0. A *p* value < 0.05 was considered statistically significant.

## 3. Results

The anthropometric and clinical characteristics of the study participants are summarized in Table 1. The analysis included a total of 139,634 individuals, comprising 83,282 men (59.6%) and 56,352 women (40.4%). The mean age of the cohort was slightly above 40 years, with the majority falling within the 30–49-year age range.

In Table 1, statistically significant differences were observed between men and women in all continuous variables (*p* < 0.001). However, due to the large sample size, effect sizes (Cohen’s *d*) were also calculated to better assess the magnitude of these differences. The largest differences were found for the height (*d* = 1.84), weight (*d* = 1.18) and systolic blood pressure (*d* = 0.67), all reflecting large or moderate effect sizes. Other variables such as age (*d* = 0.12) or total cholesterol (*d* = 0.13) showed small effect sizes, indicating limited practical significance despite statistical significance.

Table 2 shows the mean values of four insulin resistance (IR) scales by sex across sociodemographic variables and health-related behaviors. In addition to reporting statistical significance, we computed Cohen’s *d* to evaluate the magnitude of sex differences within each category. The effect sizes varied by subgroup and scale. Generally, moderate effect sizes (*d* ≈ 0.4–0.6) were observed in younger age groups (<30 and 30–39 years) and among participants with healthier lifestyles (e.g., physical activity, Mediterranean diet and no alcohol consumption), indicating meaningful sex-related differences in IR markers. The largest differences were noted in the SPISE and METS-IR scores, particularly for lifestyle-related variables, while TyG differences were more modest in most categories.

Table 3 presents the prevalence rates of high scores across the different IR risk scales included in this study. Higher values were observed with an increasing age or decreasing socioeconomic status. Additionally, elevated scores were found among individuals with unhealthy lifestyle habits, including smokers, regular alcohol consumers, sedentary individuals and those with low adherence to the Mediterranean diet. Notably, the IR risk scale scores were consistently lower among women. All observed differences were statistically significant (*p* < 0.001).

Table 4 presents the results of the binary logistic regression analysis. All independent variables included in the model (sociodemographic variables and health behaviors) were associated with high scores on the four analyzed IR risk scales. The variables showing the strongest associations (i.e., the highest odds ratios) were physical activity, adherence to the Mediterranean diet, alcohol consumption and age. Among these, the most influential modifiable variable was a lack of physical activity, with an odds ratio ranging from 8.92 to 26.52 according to the used RI risk formula.

Table 5 presents the results of the retrospective longitudinal study covering the period from 2009 (pre-period) to 2019 (post-period). Differences in the prevalence of high scores on the IR risk scales between these two periods increased with age, a decrease in socioeconomic status, or the presence of poorer health behaviors. These differences were consistently greater among men.

## 4. Discussion

According to our results, all of the analyzed variables, including both sociodemographic factors and health-related habits, were associated with the values of all of the insulin resistance (IR) scales included in the study. Among these, the most strongly associated factors were age and health-related habits such as physical activity, adherence to the Mediterranean diet and alcohol consumption.

Among the tools used to assess IR risk, notable indices included the Triglyceride-Glucose Index (TyG) [58], the Metabolic Score for Insulin Resistance (METS-IR) [59] and the Single-Point Insulin Sensitivity Estimator (SPISE) [60]. These scales have proven to be reliable predictors of insulin sensitivity and enable the early identification of individuals at risk. In this discussion, we explore how sociodemographic variables such as sex, age, social class and educational level, as well as lifestyle-related factors, influence IR and its determination through these indices.

Sex, according to our findings, was a significant determinant of insulin sensitivity and predisposition to IR. Hormonal differences [61] and body composition variations [62] between men and women influence metabolic regulation. It has been observed that men in general exhibit a higher risk of IR than women, as reflected by elevated values for the TyG and METS-IR [63].

Premenopausal women tend to demonstrate better insulin sensitivity compared with men of the same age, due to the protective effect of estrogens on glucose and lipid metabolism. However, after menopause, the decline in estrogen levels is associated with increased visceral adiposity and a higher prevalence of IR, as evidenced by significantly lower SPISE values during this stage of life [64].

Furthermore, differences in fat distribution between sexes play a key role. While men tend to accumulate fat in the visceral region, which is strongly associated with IR, women generally have a predominantly subcutaneous fat distribution, which has a lower impact on insulin sensitivity [65].

In our study, aging emerged as another determining factor in IR, likely due to the progressive decline in the body’s ability to efficiently utilize glucose with age. TyG values tend to increase with aging, reflecting a greater predisposition to impaired glucose and lipid homeostasis [66]. The METS-IR has also proven to be a useful predictor of metabolic deterioration in older populations, as it incorporates the relationship between the body mass index (BMI), triglycerides and glucose [67]. Likewise, the SPISE, an indicator of insulin sensitivity, tends to decline with age, confirming the progressive decline in metabolic efficiency over time [68].

Aging is also associated with changes in body composition, including a gradual reduction in muscle mass and an increase in abdominal fat, which contribute to a higher risk of IR [69]. Additionally, decreased physical activity with age is a worsening factor for insulin resistance, highlighting the importance of preventive strategies aimed at maintaining healthy habits in aging populations [70].

Socioeconomic status, represented in our study by social class and educational level, is associated with the values of insulin resistance (IR) risk scales. This association may be explained by the influence of these variables on multiple IR-related factors, such as access to a healthy diet, the ability to engage in regular physical activity, and exposure to risk factors like smoking and excessive alcohol consumption [71]. Epidemiological studies have shown that individuals with lower educational levels and lower social classes tend to exhibit higher values for the TyG and METS-IR, indicating a greater risk of insulin resistance in these population groups [72].

Individuals with lower education and income levels often have more limited access to healthy food options, leading to a diet high in refined sugars, saturated fats and ultra-processed foods, factors that are closely associated with IR [73]. Moreover, limited access to spaces for physical exercise and a higher prevalence of chronic stress in these groups may also contribute to dysregulated metabolic processes [74].

In contrast, individuals with higher educational attainment and better economic conditions tend to exhibit more favorable SPISE values, indicating better insulin sensitivity. This is likely due to a healthier lifestyle and greater access to preventive healthcare services [72].

Smoking was identified as an independent risk factor for IR, according to our findings. A possible explanation is that tobacco-derived products induce oxidative stress, systemic inflammation [75] and endothelial dysfunction [76]. Smokers have been observed to exhibit higher values for the TyG and METS-IR compared with non-smokers, indicating a greater risk of metabolic alterations in this population [72].

Nicotine and other tobacco compounds impair insulin sensitivity by promoting lipolysis and increasing the release of free fatty acids, contributing to the development of IR [77]. Conversely, smoking cessation has been associated with a progressive improvement in IR, emphasizing the importance of prevention strategies aimed at reducing tobacco consumption [78].

Alcohol consumption has shown a strong association with IR, according to the data obtained in this study. Some authors suggested that alcohol consumption has a dual effect on IR: while moderate consumption has been linked to improvements in insulin sensitivity [79], excessive and chronic alcohol intake has been associated with a higher risk of IR [80].

Excessive alcohol consumption promotes hepatic fat accumulation, oxidative stress and inflammation, factors that contribute to the development of IR. Additionally, alcohol can interfere with glucose homeostasis by altering hepatic glucose production and insulin secretion [81].

Diotaiuti et al. emphasized the importance of addressing both psychological and social determinants in the clinical management of addictive behaviors. Prolonged social isolation and limited interpersonal interaction can lead to significant emotional distress, often prompting individuals to seek relief through substance use or addictive behaviors, thereby perpetuating a self-reinforcing cycle. Codependency, frequently observed in clinical populations, is associated with low self-esteem, fear of abandonment, a heightened need for control, communication difficulties, indecisiveness and a diminished ability to establish personal boundaries. These traits are commonly accompanied by poor adaptability to change and an excessive need for external validation, which further increase emotional vulnerability. Impulsivity is another key component, as it impairs the capacity to delay gratification and inhibits behavioral regulation, even in the face of negative consequences. Recognizing these factors is essential for developing comprehensive and effective treatment plans that incorporate both emotional regulation and psychosocial support interventions [82].

Adherence to the Mediterranean diet has been associated with a lower risk of IR, as reflected by lower values for the TyG, METS-IR, and SPISE-IR. This dietary pattern, rich in monounsaturated fatty acids, antioxidants and fiber, contributes to improved insulin sensitivity and reduced systemic inflammation, as reported in previous studies [82].

Moreover, our findings indicate that regular physical activity is one of the most protective factors against IR. Exercise enhances glucose uptake by skeletal muscle, increases insulin sensitivity and reduces triglyceride levels [83]. Studies have shown that physically active individuals exhibit significantly lower TyG, METS-IR, and SPISE-IR values compared with sedentary individuals [84].

### Strengths and Limitations

The most significant strength of this study is the large sample size, which included a population of 139,634 Spanish workers from various regions and occupational sectors. This enhanced the representativeness, statistical power and generalizability of the study’s findings to the working population at large.

Another strength lies in the use of three validated indices (TyG, METS-IR and SPISE) to assess insulin resistance, allowing for a more comprehensive evaluation of metabolic health. Its dual-phase design—cross-sectional and retrospective longitudinal—combined with robust statistical analyses such as binary logistic regression strengthened the reliability of the observed associations.

This study also stands out for the collection of standardized anthropometric, biochemical and lifestyle data, which minimized measurement error and variability. By accounting for multiple sociodemographic and lifestyle factors, the research provides a comprehensive view of the determinants of insulin resistance. Furthermore, the use of validated questionnaires on physical activity and adherence to the Mediterranean diet enhanced the reliability and feasibility of both the study and its longitudinal follow-up.

Finally, the study’s focus on occupational health offers valuable insights into metabolic risk factors among the working-age population, an often underrepresented group in metabolic health research.

On the other hand, the cross-sectional design limits the ability to establish causal relationships, as data are collected at a single point in time, making it difficult to distinguish between cause and effect. In addition, selection bias may occur if the sample does not adequately represent the population, and there is a risk of confounding due to external variables. Nevertheless, the large sample size partially mitigates these limitations. The use of self-administered questionnaires may introduce recall bias or social desirability bias. To improve validity, future studies are encouraged to incorporate objective tools such as pedometers and detailed dietary records.

Since the study population was drawn from individuals undergoing occupational health evaluations, there is an important limitation regarding the representativeness of the results. This methodology may exclude unemployed individuals, those outside the active working age range and those with severe health conditions that prevent them from working. Therefore, these factors limit the generalizability of the findings to the general population.

Another limitation of the study is the absence of direct insulin measurements, relying solely on indirect markers (TyG, METS-IR, and SPISE), which may have affected the accuracy of the assessment. Furthermore, the METS-IR and SPISE may be influenced by individual metabolic variations, ethnic factors, and inflammatory states, reducing their precision in certain populations, particularly among individuals with severe obesity. This study also presents a limited temporal resolution; although it included a longitudinal component (2009–2019), it did not allow for continuous monitoring of changes in behavior or metabolic health over time.

Finally, this study did not differentiate between types of alcoholic beverages or consumption patterns (excessive versus regular), which could influence metabolic outcomes. Additionally, it lacked information on genetic predisposition or inflammatory markers, factors that are relevant for a more accurate assessment of insulin resistance risk. The absence of these data limits comprehensive understanding of the impact of alcohol consumption and other biological factors on the development of metabolic alterations.

## 5. Conclusions

Sociodemographic variables and health-related habits play a crucial role in insulin resistance (IR), influencing its development and progression. The implementation of preventive strategies and the promotion of healthy lifestyles are essential to reducing metabolic risk and improving insulin sensitivity, particularly in vulnerable populations.

## Figures and Tables

**Table 1:** Characteristics of the population, with means, standard deviations and effect sizes (Cohen’s *d*) for each comparison between men and women.

	Men’s Mean (SD) n = 83,282	Women’s Mean (SD) n = 56,352	Cohen’s *d*
Age (years)	41.4 (10.7)	40.1 (10.4)	0.123
Height (cm)	173.8 (7.1)	161.2 (6.5)	1.836
Weight (kg)	83.2 (14.6)	66.3 (13.9)	1.18
Systolic blood pressure (mmHg)	126.2 (15.9)	115.6 (15.7)	0.67
Diastolic blood pressure (mmHg)	76.6 (10.9)	71.1 (10.7)	0.508
Total cholesterol (mg/dL)	199.6 (38.6)	194.6 (36.9)	0.132
HDL cholesterol (mg/dL)	50.0 (7.7)	54.7 (9.2)	−0.564
LDL cholesterol (mg/dL)	122.6 (37.4)	121.5 (37.1)	0.03
Triglycerides (mg/dL)	133.8 (95.6)	90.8 (49.7)	0.536
Glucose (mg/dL)	93.0 (25.4)	86.8 (18.1)	0.273
	Cramers_V		
<30 years vs. ≥30 years	0.038512766469072986		
Social class I vs. other social class	0.10030239261755346		
Elementary school vs. higher education	0.18254337547869515		
Non-smokers vs. smokers	0.011487974483581643		
No physical activity vs. physical activity	0.10931382468740578		
Non-Meditrerranean diet vs. Mediterranean diet	0.13048756330861092		
No alcohol consumption vs. alcohol consumption	0.19172611388544775		

**Table 2:** Mean values of four insulin resistance (IR) scales by sex across sociodemographic variables and health-related behaviors.

	n	Scale	Men	n	Scale	Women	Cohen’s *d*
			Mean (SD)			Mean (SD)	
<30 years	12,558	TyG	8.2 (0.5)	10,110	TyG	8.0 (0.4)	0.437
<30 years	12,558	TyG-BMI	207.4 (42.4)	10,110	TyG-BMI	189.4 (42.0)	0.426
<30 years	12,558	METS-IR	37.4 (7.8)	10,110	METS-IR	33.8 (7.6)	0.467
<30 years	12,558	SPISE-IR	1.5 (0.4)	10,110	SPISE-IR	1.3 (0.4)	0.500
30–39 years	24,648	TyG	8.4 (0.6)	17,460	TyG	8.1 (0.5)	0.535
30–39 years	24,648	TyG-BMI	227.6 (45.6)	17,460	TyG-BMI	199.0 (47.1)	0.619
30–39 years	24,648	METS-IR	41.2 (8.5)	17,460	METS-IR	35.8 (8.6)	0.632
30–39 years	24,648	SPISE-IR	1.7 (0.5)	17,460	SPISE-IR	1.4 (0.5)	0.600
40–49 years	25,178	TyG	8.6 (0.6)	17,094	TyG	8.2 (0.5)	0.712
40–49 years	25,178	TyG-BMI	243.5 (46.9)	17,094	TyG-BMI	214.6 (49.8)	0.601
40–49 years	25,178	METS-IR	44.3 (8.8)	17,094	METS-IR	38.7 (9.1)	0.628
40–49 years	25,178	SPISE-IR	1.9 (0.5)	17,094	SPISE-IR	1.6 (0.5)	0.600
50–59 years	17,370	TyG	8.8 (0.6)	9984	TyG	8.4 (0.5)	0.707
50–59 years	17,370	TyG-BMI	253.9 (45.7)	9984	TyG-BMI	232.6 (51.3)	0.445
50–59 years	17,370	METS-IR	46.6 (8.7)	9984	METS-IR	41.9 (9.3)	0.527
50–59 years	17,370	SPISE-IR	2.0 (0.5)	9984	SPISE-IR	1.7 (0.5)	0.600
60–69 years	3528	TyG	8.9 (0.5)	1704	TyG	8.5 (0.5)	0.800
60–69 years	3528	TyG-BMI	260.2 (41.5)	1704	TyG-BMI	241.8 (47.6)	0.422
60–69 years	3528	METS-IR	48.0 (8.0)	1704	METS-IR	43.5 (8.6)	0.549
60–69 years	3528	SPISE-IR	2.1 (0.5)	1704	SPISE-IR	1.8 (0.5)	0.600
Social class I	6234	TyG	8.4 (0.6)	7632	TyG	8.0 (0.4)	0.893
Social class I	6234	TyG-BMI	229.3 (43.3)	7632	TyG-BMI	191.3 (41.4)	0.901
Social class I	6234	METS-IR	41.9 (8.3)	7632	METS-IR	34.0 (7.7)	1.027
Social class I	6234	SPISE-IR	1.7 (0.5)	7632	SPISE-IR	1.3 (0.4)	1.000
Social class II	19,856	TyG	8.5 (0.6)	18,112	TyG	8.1 (0.5)	0.721
Social class II	19,856	TyG-BMI	236.8 (45.6)	18,112	TyG-BMI	202.0 (46.1)	0.751
Social class II	19,856	METS-IR	43.0 (8.7)	18,112	METS-IR	36.3 (8.4)	0.784
Social class II	19,856	SPISE-IR	1.8 (0.5)	18,112	SPISE-IR	1.4 (0.5)	0.800
Social class III	57,192	TyG	8.5 (0.7)	30,608	TyG	8.2 (0.5)	0.529
Social class III	57,192	TyG-BMI	239.5 (49.3)	30,608	TyG-BMI	218.0 (52.8)	0.449
Social class III	57,192	METS-IR	43.1 (9.3)	30,608	METS-IR	39.3 (9.6)	0.427
Social class III	57,192	SPISE-IR	1.8 (0.5)	30,608	SPISE-IR	1.6 (0.5)	0.400
Elementary school	55,306	TyG	8.7 (0.6)	27,086	TyG	8.2 (0.8)	0.879
Elementary school	55,306	TyG-BMI	234.8 (47.8)	27,086	TyG-BMI	218.0 (52.0)	0.353
Elementary school	55,306	METS-IR	43.8 (9.3)	27,086	METS-IR	39.3 (9.5)	0.506
Elementary school	55,306	SPISE-IR	1.9 (0.5)	27,086	SPISE-IR	1.6 (0.5)	0.600
High school	22,408	TyG	8.6 (0.7)	22,574	TyG	8.1 (0.5)	1.000
High school	22,408	TyG-BMI	231.8 (49.5)	22,574	TyG-BMI	204.5 (48.6)	0.611
High school	22,408	METS-IR	42.8 (9.0)	22,574	METS-IR	36.7 (8.8)	0.734
High school	22,408	SPISE-IR	1.8 (0.5)	22,574	SPISE-IR	1.5 (0.5)	0.600
University	5568	TyG	8.5 (0.6)	6692	TyG	8.0 (0.4)	1.116
University	5568	TyG-BMI	230.4 (43.1)	6692	TyG-BMI	190.0 (40.3)	0.958
University	5568	METS-IR	42.3 (8.3)	6692	METS-IR	33.8 (7.5)	1.105
University	5568	SPISE-IR	1.7 (0.5)	6692	SPISE-IR	1.3 (0.4)	1.000
Non-smokers	55,618	TyG	8.5 (0.6)	38,252	TyG	8.1 (0.5)	0.712
Non-smokers	55,618	TyG-BMI	229.9 (49.1)	38,252	TyG-BMI	203.9 (47.6)	0.563
Non-smokers	55,618	METS-IR	42.1 (9.5)	38,252	METS-IR	36.7 (8.7)	0.632
Non-smokers	55,618	SPISE-IR	1.7 (0.6)	38,252	SPISE-IR	1.4 (0.5)	0.600
Smokers	27,664	TyG	8.6 (0.7)	18,100	TyG	8.2 (0.5)	0.711
Smokers	27,664	TyG-BMI	239.3 (47.3)	18,100	TyG-BMI	211.8 (51.3)	0.572
Smokers	27,664	METS-IR	43.5 (8.8)	18,100	METS-IR	38.1 (9.4)	0.605
Smokers	27,664	SPISE-IR	1.8 (0.5)	18,100	SPISE-IR	1.5 (0.5)	0.600
No physical activity	51,984	TyG	8.8 (0.6)	28,962	TyG	8.4 (0.5)	0.706
No physical activity	51,984	TyG-BMI	260.7 (42.7)	28,962	TyG-BMI	240.9 (49.7)	0.414
No physical activity	51,984	METS-IR	47.6 (8.1)	28,962	METS-IR	43.5 (9.0)	0.460
No physical activity	51,984	SPISE-IR	2.1 (0.5)	28,962	SPISE-IR	1.8 (0.5)	0.600
Physical activity	31,298	TyG	8.1 (0.4)	27,390	TyG	7.9 (0.4)	0.400
Physical activity	31,298	TyG-BMI	195.6 (21.8)	27,390	TyG-BMI	175.8 (20.4)	0.445
Physical activity	31,298	METS-IR	35.3 (3.9)	27,390	METS-IR	31.4 (3.7)	0.471
Physical activity	31,298	SPISE-IR	1.4 (0.2)	27,390	SPISE-IR	1.2 (0.2)	0.400
Non-Mediterranean diet	54,792	TyG	8.8 (0.6)	29,764	TyG	8.4 (0.5)	0.856
Non-Mediterranean diet	54,792	TyG-BMI	257.4 (44.2)	29,764	TyG-BMI	237.7 (51.6)	0.466
Non-Mediterranean diet	54,792	METS-IR	46.9 (8.4)	29,764	METS-IR	42.8 (9.4)	0.530
Non-Mediterranean diet	54,792	SPISE-IR	2.0 (0.5)	29,764	SPISE-IR	1.8 (0.5)	0.500
Mediterranean diet	28,490	TyG	8.1 (0.4)	26,588	TyG	7.9 (0.4)	0.361
Mediterranean diet	28,490	TyG-BMI	195.4 (22.0)	26,588	TyG-BMI	177.4 (21.6)	0.389
Mediterranean diet	28,490	METS-IR	35.4 (4.0)	26,588	METS-IR	31.8 (4.0)	0.421
Mediterranean diet	28,490	SPISE-IR	1.4 (0.2)	26,588	SPISE-IR	1.2 (0.2)	0.400
No alcohol consumption	56,022	TyG	8.4 (0.5)	47,536	TyG	8.1 (0.4)	0.539
No alcohol consumption	56,022	TyG-BMI	217.4 (37.6)	47,536	TyG-BMI	197.6 (40.2)	0.410
No alcohol consumption	56,022	METS-IR	39.5 (7.1)	47,536	METS-IR	35.5 (7.4)	0.447
No alcohol consumption	56,022	SPISE-IR	1.6 (0.4)	47,536	SPISE-IR	1.4 (0.4)	0.400
Alcohol consumption	27,260	TyG	8.9 (0.7)	8816	TyG	8.6 (0.6)	0.520
Alcohol consumption	27,260	TyG-BMI	274.8 (44.0)	8816	TyG-BMI	272.2 (52.5)	0.055
Alcohol consumption	27,260	METS-IR	50.2 (8.4)	8816	METS-IR	48.9 (9.7)	0.147
Alcohol consumption	27,260	SPISE-IR	2.2 (0.5)	8816	SPISE-IR	2.1 (0.6)	0.200

TyG = Triglyceride-Glucose Index; BMI = body mass index; METS-IR = Metabolic Score for Insulin Resistance; SPISE-IR = Single-Point Insulin Sensitivity Estimator for Insulin Resistance; SD = standard deviation.

**Table 3:** Prevalence of high values of different IR scales according sociodemographic variables and healthy habits by sex.

		TyG Index High	TyG-BMI High	METS-IR High	SPISE-IR High
**Men**	**n**	**%**	**%**	**%**	**%**
<30 years	12,558	11.6	12.8	7.1	28.5
30–39 years	24,648	22.6	22.3	13.9	47.9
40–49 years	25,178	36.1	35.8	22.8	64.7
50–59 years	17,370	45.8	45.3	31.4	74.7
60–69 years	3528	55.8	51.6	36.6	82.3
Social class I	6234	24.5	24.2	16.2	51.8
Social class II	19,856	31.1	30.7	19.4	57.3
Social class III	57,192	32.1	31.8	20.9	58.8
Elementary school	55,306	36.0	34.0	22.5	59.3
High school	22,408	29.9	30.4	19.6	56.5
University	5568	25.8	25.3	17.1	54.2
Non-smokers	55,618	30.3	26.7	18.6	51.3
Smokers	27,664	33.2	33.1	21.0	60.0
No physical activity	51,984	47.8	49.6	32.4	85.2
Physical activity	31,298	3.8	3.9	4,1	10.5
Non-Mediterranean diet	54,792	45.3	47.0	30.7	81.0
Mediterranean diet	28,490	4.4	5.8	5.9	11.2
No alcohol consumption	56,022	20.1	14.5	7.8	41.0
Alcohol consumption	27,260	54.1	64.8	45.5	90.3
**Women**	**n**	**%**	**%**	**%**	**%**
<30 years	10,110	5.8	8.0	4.5	16.2
30–39 years	17,460	8.1	11.9	7.5	22.1
40–49 years	17,094	14.3	17.7	11.3	34.1
50–59 years	9984	25.7	27.7	17.7	50.4
60–69 years	1704	37.1	35.2	23.8	60.9
Social class I	7632	7.4	7.6	4.5	16.5
Social class II	18,112	11.9	12.2	7.6	24.4
Social class III	30,608	16.1	21.2	13.6	38.3
Elementary school	27,086	15.8	21.0	13.3	38.6
High school	22,574	12.8	13.9	8.9	26.1
University	6692	7.2	6.8	4.3	15.5
Non-smokers	38,252	13.5	13.2	8.3	26.8
Smokers	18,100	13.8	18.8	11.5	32.8
No physical activity	28,962	25.5	32.0	20.3	59.7
Physical activity	27,390	2.8	2.5	1.9	7.8
Non-Mediterranean diet	29,764	24.1	31.2	19.8	57.2
Mediterranean diet	26,588	3.1	3.8	2.9	10.3
No alcohol consumption	47,536	7.9	8.8	4.8	21.7
Alcohol consumption	8816	44.4	57.9	40.9	80.5

TyG = Triglyceride-Glucose Index; BMI = body mass index; METS-IR = Metabolic Score for Insulin Resistance; SPISE-IR = Single-Point Insulin Sensitivity Estimator for Insulin Resistance.

**Table 4:** Binary logistic regression.

	TyG Index High n = 33,702	TyG-BMI High n = 35,088	METS-IR High n = 22,704	SPISE-IR High n = 74,686
	OR (95% CI)	OR (95% CI)	OR (95% CI)	OR (95% CI)
Women	1	1	1	1
Men	2.41 (2.34–2.49)	1.48 (1.44–1.53)	1.33 (1.28–1.38)	3.63 (3.51–3.75)
<30 years	1	1	1	1
30–39 years	1.48 (1.39–1.58)	1.10 (1.07–1.13)	1.11 (1.08–1.14)	1.40 (1.29–1.51)
40–49 years	1.81 (1.69–1.93)	1.19 (1.13–1.25)	1.18 (1.13–1.24)	1.47 (1.38–1.57)
50–59 years	2.43 (2.26–2.60)	1.39 (1.28–1.50)	1.38 (1.25–1.51)	1.76 (1.60–1.92)
60–69 years	3.39 (3.12–3.66)	1.76 (1.51–2.02)	1.80 (1.60–2.01)	2.45 (2.22–2.69)
Social class I	1	1	1	1
Social class II	1.51 (1.45–1.58)	1.39 (1.27–1.50)	1.52 (1.45–1.60)	1.50 (1.39–1.61)
Social class III	1.97 (1.67–2.27)	1.58 (1.50–1.65)	1.67 (1.57–1.78)	1.88 (1.62–2.14)
University	1	1	1	1
High school	1.38 (1.27–1.49)	1.21 (1.15–1.26)	1.45 (1.33–1.58)	1.39 (1.30–1.49)
Elementary school	1.95 (1.78–2.13)	1.65 (1.50–1.81)	1.76 (1.60–1.93)	1.79 (1.60–1.98)
Non-smokers	1	1	1	1
Smokers	1.19 (1.14–1.25)	1.20 (1.13–1.28)	1.18 (1.12–1.24)	1.06 (1.03–1.10)
No physical activity	1	1	1	1
Physical activity	11.21 (10.30–12.12)	13.74 (12.70–14.80)	8.92 (7.93–9.94)	16.52 (15.57–16.48)
Mediterranean diet	1	1	1	1
Non-Mediterranean diet	1.64 (1.51–1.78)	5.46 (4.90–6.03)	3.22 (2.80–3.65)	3.04 (2.86–3.23)
No alcohol consumption	1	1	1	1
Alcohol consumption	2.43 (2.35–2.51)	5.06 (4.89–5.23)	5.06 (4.87–5.25)	4.00 (3.83–4.17)

TyG = Triglyceride-Glucose Index; BMI = body mass index; METS-IR = Metabolic Score for Insulin Resistance; SPISE-IR = Single-Point Insulin Sensitivity Estimator for Insulin Resistance; OR = odds ratio.

**Table 5:** Longitudinal retrospective study by sex.

		TyG High			TyG-BMI High			METS-IR High			SPISE-IR High		
		PRE	POST		PRE	POST		PRE	POST		PRE	POST	
**Men**	**n**	**%**	**%**	**Difference (%)**	**%**	**%**	**Difference (%)**	**%**	**%**	**Difference (%)**	**%**	**%**	**Difference (%)**
<30 years	3645	10.7	11.7	9.3	11.8	13.1	11.0	6.9	7.7	11.6	27.3	29.2	7.0
30–39 years	6933	21.9	25	14.2	22	25	13.6	13.9	15.8	13.7	46.9	51.1	9.0
40–49 years	7013	35.4	42.1	18.9	35.3	41.5	17.6	22.4	26.4	17.9	64.8	71.8	10.8
50–59 years	4952	46.2	56.5	22.3	46.9	57.2	22.0	32.4	40.1	23.8	75.2	85.6	13.8
Social class I	1760	23.4	25.8	10.3	24	26.6	10.8	16.3	18.2	11.7	50.3	54.3	8.0
Social class II	5368	30.9	36.6	18.4	30.9	36.4	17.8	19.5	23.1	18.5	57	63.6	11.6
Social class III	15,415	31.8	38.6	21.4	31.7	38.1	20.2	26.6	32.2	21.1	58.3	67.1	15.1
Elementary school	14,914	36.2	43.8	21.0	34.4	41.4	20.3	22.6	27.6	22.1	59.7	68.5	14.7
High school	6053	29.5	35	18.6	30.1	35.5	17.9	19.6	23.2	18.4	56.1	62.5	11.4
University	1576	23.8	26.2	10.1	25	27.8	11.2	17	19.1	12.4	52.4	56.7	8.2
Non-smokers	15,122	30.1	35.8	18.9	26.3	31	17.9	18.6	22.2	19.4	50.6	56.1	10.9
Smokers	7421	32.6	41	25.8	33.2	42.2	27.1	21.1	25.6	21.3	59.8	68.9	15.2
Physical activity	8535	3.8	4	5.3	3.9	4.2	7.7	3.9	4.2	7.7	10.1	10.7	5.9
No physical activity	14,008	47.5	59.8	25.9	49.8	60.9	22.3	32.6	40.3	23.6	85.2	94.3	10.7
Mediterranean diet	7767	4.2	4.5	7.1	5.9	6.4	8.5	4.8	5.2	8.3	10.7	11.4	6.5
Non-Mediterranean diet	14,776	45	56.2	24.9	47.2	57.2	21.2	30.9	37.8	22.3	81	93.6	15.6
No alcohol consumption	15,107	19.6	21.6	10.2	14.3	16.6	16.1	7.9	9.2	16.5	40.1	44.2	10.2
Alcohol consumption	7436	53.9	70	29.9	64.7	80.1	23.8	45.3	62.2	37.3	90.6	97	7.1
**Women**	**n**	**%**	**%**	**Difference (%)**	**%**	**%**	**Difference (%)**	**%**	**%**	**Difference (%)**	**%**	**%**	**Difference (%)**
< 30 years	2833	5.9	6.4	8.5	8.7	9.5	9.2	5	5.5	10.0	17.4	18.9	8.6
30–39 years	4824	8.6	9.7	12.8	12.3	13.8	12.2	7.7	8.6	11.7	22.2	25	12.6
40–49 years	4636	14.3	17.1	19.6	16.8	19.5	16.1	10.8	12.6	16.7	34.1	39.1	14.7
50–59 years	2768	26.2	32.3	23.3	27.5	34.9	26.9	18.1	22.3	23.2	49.5	58.4	18.0
Social class I	1973	8	8.7	8.7	7.1	7.7	8.5	3.9	4.3	10.3	16.6	17.7	6.6
Social class II	4920	11.9	13.4	12.6	12.2	13.8	13.1	7.8	9.1	16.7	24.2	37.7	55.8
Social class III	8168	16.3	19.6	20.2	21.1	25.4	20.4	13.7	16.8	22.6	38.7	45.1	16.5
Elementary school	7289	16	19.2	20.0	20.9	25.2	20.6	13.4	16.5	23.1	38.8	45.4	17.0
High school	6056	12.8	14.5	13.3	13.8	15.6	13.0	8.9	10.3	15.7	26.2	28.8	9.9
University	1716	7.9	8.7	10.1	6.2	6.7	8.1	3.8	4.2	10.5	15.4	16.5	7.1
Non-smokers	10,236	13.4	15.4	14.9	12.6	14.3	13.5	7.8	8.7	11.5	27.3	30.2	10.6
Smokers	4825	14	16.4	17.1	18.2	21.5	18.1	11.8	13.7	16.1	32.9	38.5	17.0
Physical activity	7317	1.2	1.3	8.3	3.2	3.3	3.1	1.9	2	5.3	7.9	8.4	6.3
No physical activity	7744	25.7	31	20.6	31.8	40.9	28.6	20.4	24.5	20.1	60.1	73.8	22.8
Mediterranean diet	7029	1.9	2	5.3	4.8	5.1	6.3	2.8	3	7.1	8.8	9.5	8.0
Non-Mediterranean diet	8032	24.2	29.7	22.7	30.7	39.9	30.0	19.7	23.6	19.8	57.2	68.8	20.3
No alcohol consumption	12,750	8.2	9	9.8	8.9	10	12.4	4.9	5.4	10.2	22.1	25.3	14.5
Alcohol consumption	2311	44.8	58.1	29.7	57.8	74.7	29.2	41.1	52	26.5	80.5	94.8	17.8

TyG = Triglyceride-Glucose Index; BMI = body mass index; METS-IR = Metabolic Score for Insulin Resistance; SPISE-IR = Single-Point Insulin Sensitivity Estimator for Insulin Resistance; PRE = year 2009, POST = year 2019. The formula for calculating the difference is [POST − PRE/PRE] as a percentage.

## Data Availability

The data collected in this study are securely stored in a database that complies with all security measures at ADEMA-Escuela Universitaria. The Data Protection Officer overseeing this is Ángel Arturo López González.
